# Cellular Effects of T-2 Toxin on Primary Hepatic Cell Culture Models of Chickens

**DOI:** 10.3390/toxins12010046

**Published:** 2020-01-13

**Authors:** Máté Mackei, Kata Orbán, Andor Molnár, László Pál, Károly Dublecz, Ferenc Husvéth, Zsuzsanna Neogrády, Gábor Mátis

**Affiliations:** 1 Division of Biochemistry, Department of Physiology and Biochemistry, University of Veterinary Medicine, István utca 2, H-1078 Budapest, Hungary; Orban.Kata@univet.hu (K.O.); Neogrady.Zsuzsanna@univet.hu (Z.N.); Matis.Gabor@univet.hu (G.M.); 2 Department of Animal Science, Georgikon Faculty, University of Pannonia, Deák Ferenc utca 16, H-8360 Keszthely, Hungary; andor.molnar@georgikon.hu (A.M.); pal-l@georgikon.hu (L.P.); dublecz@georgikon.hu (K.D.); hferenc48@georgikon.hu (F.H.)

**Keywords:** mycotoxin, trichothecenes, T-2 toxin, hepatocyte, broiler chickens, heat shock proteins, oxidative stress, interleukins

## Abstract

Trichothecene mycotoxins such as T-2 toxin cause severe problems for agriculture, as well as for veterinary medicine. As liver is one of the key organs in metabolism, the main aim of our study was to investigate the immunomodulatory and cytotoxic effects of T-2 toxin, using primary hepatocyte mono-culture and hepatocyte—nonparenchymal cell (predominantly Kupffer cell) co-culture models of chicken. Cultures were exposed to 10 (T10 group), 100 (T100 group) and 1000 (T1000 group) nmol/L T-2 toxin treatment for 8 or 24 h. Alterations of cellular metabolic activity, the production of reactive oxygen species (extracellular H_2_O_2_), heat shock protein 70 (HSP70), and the concentration of different inflammatory cytokines such as interleukin (IL-)6 and IL-8 were investigated. Metabolic activity was intensely decreased by T-2 toxin administration in all of the cell culture models, in every applied concentration and incubation time. Concentrations of HSP70 and IL-8 were significantly increased in hepatocyte mono-cultures exposed to higher T-2 toxin levels (both in T100 and T1000 groups for HSP70 and in T1000 group for IL-8, respectively) compared to controls after 24 h incubation. Similarly, IL-6 levels were also significantly elevated in the T100 and T1000 groups in both of mono- and co-cultures, but only after 8 h of incubation time. In spite of the general harmful effects of T-2 toxin treatment, no significant differences were observed on reactive oxygen species production. Furthermore, the two cell culture models showed different levels of H_2_O_2_, HSP70, and IL-8 concentrations independently of T-2 toxin supplementation. In conclusion, the established primary cell cultures derived from chicken proved to be proper models to study the specific molecular effects caused by T-2 toxin. Metabolic activity and immune status of the different examined cell cultures were intensively affected; however, no changes were found in H_2_O_2_ levels.

## 1. Introduction

Mycotoxin contamination of food and feed has an exceptional importance worldwide. Trichothecenes, as remarkable secondary metabolites of various *Fusarium* species, had received great attention nowadays. They naturally occur in mouldy grains, infecting the plants already on the field before harvesting [1]. The T-2 toxin is one of the most noxious members of the trichothecene mycotoxin group [2]. Contamination caused by T-2 toxin was shown to be a serious hazard for general food and feed safety and in consequence, for human and animal health [3].

Trichothecenes are mycotoxins, having a sesquiterpene skeleton. The 12,13-epoxy ring structure is important for toxic effects and biological activity. Based on different substitutions at five positions of the structure, they can be chemically classified into four types. The T-2 belongs to group A trichothecenes [4]. According to recent surveys, depending on different regions, approximately 40%–60% of main commodities contained various mycotoxins above risk threshold in Europe. Among these samples, 20%–40% of feed samples contained hazardous T-2 toxin levels [5]. As one of the most toxic mycotoxins, it is in the focus of numerous in vivo and in vitro toxicological studies. However, there are still a lot of questions open, in relation to its mechanism of action or molecular effects.

Regarding metabolism, T-2 toxin has a lipophilic character and can be immediately absorbed from the gastrointestinal tract or through the respiratory mucosal membranes [6]. After absorption, liver is the primary organ of its metabolism. Major metabolic pathways of T-2 toxin are hydrolysis, hydroxylation, and conjugation. De-epoxidation is also a crucial step in the detoxification of trichothecenes [7]. Half-life of T-2 toxin in blood plasma is mostly short, and elimination is usually completed within 48 h, depending on the application mode, the ingested amount and species-specific differences. After exposure, the ingested toxin does not accumulate to a significant quantity in the different organs such as the liver, the kidney, or the skeletal muscle [8,9].

At cellular level, one of the main harmful effects of T-2 toxin are the primary inhibition of protein synthesis by binding peptidyl transferase enzyme and causing disorders in the translation, targeting the 60S ribosomal subunit [6,10]. Additionally, the presence of the toxin can lead to disturbances on the genetic level as well, such as the induction of DNA fragmentation or DNA lesions [11].

Another highly important nonribosomal effect of T-2 toxin is the intensive free radical production and the oxidative stress associated harmful effects, such as nuclear and mitochondrial DNA damage, elevated lipid peroxidation and disturbances in the cell signalling and inflammatory pathways [12,13,14]. In most cases, T-2 toxin significantly increases the level of reactive oxygen species (ROS) and induces changes in the antioxidant status of the cells [11,15], while in other studies beside the intensive cellular damage oxidative stress was not detected [16,17].

Furthermore, on cellular level, the 70 kilodalton heat shock protein (HSP70) is one of the most important member of the HSP family and the expression of this protein shows correlation with the cytoprotective mechanisms against different toxic effects [18]. Data regarding the effects of trichothecenes on influencing HSP70 gene expression are limited. Elevated HSP70 expression caused by T-2 toxin was observed in the placenta of pregnant rat in vivo [19] and similarly, T-2 toxin induced HSP70 protein production in vitro in Vero cells [20].

Regarding the effects of T-2 toxin on cytokines and on other inflammatory mediators, conflicting results can be found in the literature. In vitro studies described reduced interleukin (IL)-1β and tumor necrosis factor (TNF)-α concentrations in primary porcine macrophages [21], while other experiments suggested, that together with lipopolysaccharides, the toxin synergistically activated IL-1β and IL-18 mediated inflammatory response in human macrophages in vitro [22].

Avian species are relatively tolerant to trichothecenes in comparison with mammals; however, the presence of T-2 toxin in the feed serves as a relevant problem in poultry industry worldwide. This decreased sensitivity is based on the moderate absorption after oral exposure, the extensive metabolism and rapid elimination of trichothecenes in bird species [23]. Although several studies exist about the effects of T-2 toxin in various poultry species, there are numerous questions regarding the mode of action on the molecular level and considering the species-specific differences in the effects of the toxin.

Therefore, the main aim of the present study was to investigate the cellular effects of T-2 toxin on different hepatic cell culture models of chicken origin in vitro. In cell cultures the chemical and physiological factors of the microenvironment can be precisely controlled, so the effects of T-2 toxin can be more accurately studied on cellular level. As playing a crucial role in the detoxification processes, and as one of the main targets of the trichothecenes, liver is especially exposed to the harmful effects of T-2 toxin. Further, as other studies provided different and often conflicting results depending on the investigated organs or cell types, another goal of the trial was to compare the effects of T-2 toxin on primary liver-derived hepatocyte mono-cultures and hepatocyte—nonparenchymal cell (NP cell; predominantly resident macrophages, Kupffer cells) co-cultures of chicken origin in vitro. According to the hypothesis of our study, T-2 toxin may alter metabolic activity, redox status, and pro-inflammatory cytokine (IL-6 and IL-8) production of the examined cell culture models.

## 2. Results

### 2.1. Cellular Metabolic Activity

**Figure 1:**
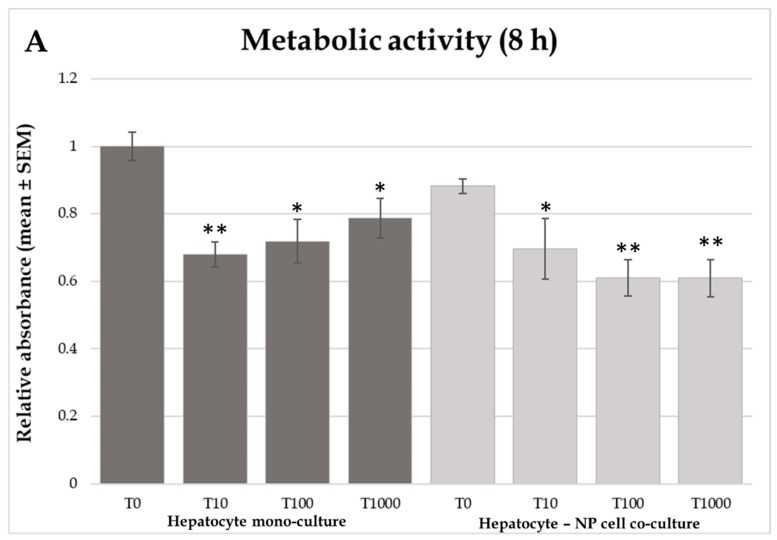

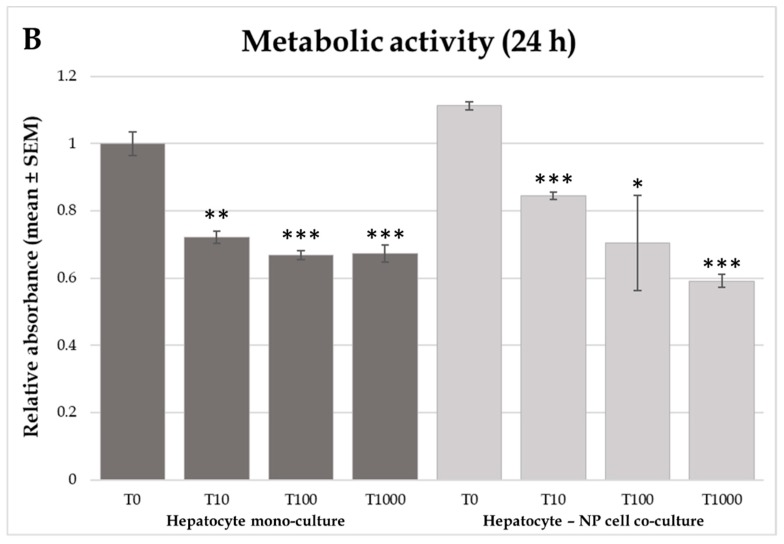
Effects of T-2 toxin treatment on cellular metabolic activity of chicken-derived primary hepatocyte mono- and hepatocyte—nonparenchymal cell co-cultures with the cell ratio of 6:1 (hepatocytes to nonparenchymal cells). Cells after 24 h culturing were treated with different concentrations of T-2 toxin for 8 (**A**) or 24 h (**B**). “T0” refers to control group (without T-2 toxin treatment); “T10” refers to 10 nmol/L T-2 toxin treatment; “T100” refers to 100 nmol/L T-2 toxin treatment; T1000 refers to 1000 nmol/L T-2 toxin treatment. Cellular metabolic activity was measured by Cell Counting Kit-8 (CCK-8) assay. Relative absorbances were calculated by considering the mean value of T0 hepatocyte mono-cultures as 1. Results are expressed as mean ± SEM. * *p* < 0.05; ** *p* < 0.01; *** *p* < 0.001. n = 3/group.

The T-2 toxin treatment decreased the metabolic activity of cultured cells at all applied T-2 toxin concentrations both in the hepatocyte mono-culture and in the hepatocyte-NP cell co-culture models, following both 8 (Figure 1A) and 24 h (Figure 1B) incubation times (8 h incubation of hepatocyte mono-culture: *p* = 0.00299; *p* = 0.011; *p* = 0.0272 for 10, 100, and 1000 nmol/L T-2 toxin, respectively; 8 h incubation of co-culture: *p* = 0.0454; *p* = 0.00201; *p* = 0.00214 for 10, 100, and 1000 nmol/L T-2 toxin, respectively, 24 h incubation of hepatocyte mono-culture: *p* = 0.00204; *p* < 0.001; *p* = 0.00154 for 10, 100, and 1000 nmol/L T-2 toxin, respectively, 24 h incubation of co-culture: *p* < 0.001; *p* = 0.0434; *p* < 0.001 for 10, 100, and 1000 nmol/L T-2 toxin, respectively).

### 2.2. H_2_O_2_ Production

**Figure 2:**
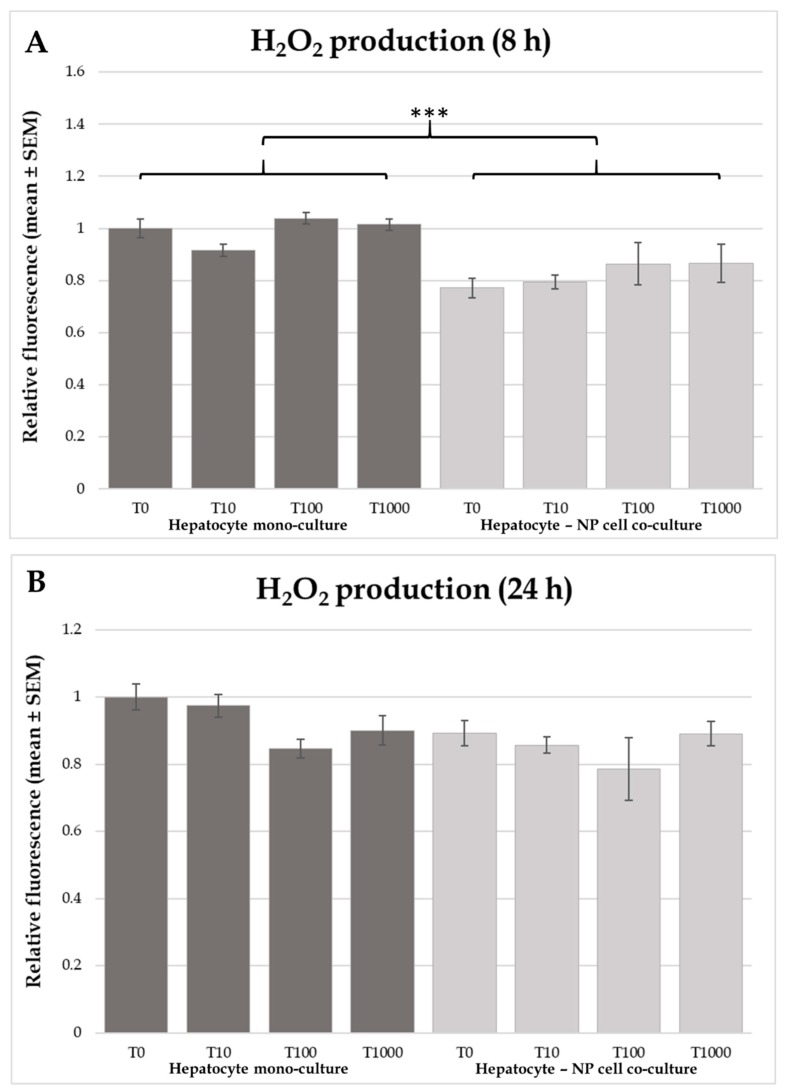
Effects of T-2 toxin treatment on H_2_O_2_ production of chicken-derived primary hepatocyte mono- and hepatocyte—nonparenchymal cell co-cultures with the cell ratio of 6:1 (hepatocytes to nonparenchymal cells). Cells after 24 h culturing were treated with different concentrations of T-2 toxin for 8 (**A**) or 24 h (**B**). “T0” refers to control group (without T-2 toxin treatment); “T10” refers to 10 nmol/L T-2 toxin treatment; “T100” refers to 100 nmol/L T-2 toxin treatment; T1000 refers to 1000 nmol/L T-2 toxin treatment. H_2_O_2_ production was measured by the Amplex Red method. Relative fluorescences were calculated by considering the mean value of T0 hepatocyte mono-cultures as 1. Results are expressed as mean ± SEM. *** *p* < 0.001. n = 3/group.

According the present results, H_2_O_2_ production was not affected by T-2 toxin treatment using 8 h or 24 h incubation in any of the applied cell culture models (Figure 2). Comparing the culture specific differences, a higher H_2_O_2_ production rate was observed in the hepatocyte mono-culture model than in the co-culture after 8 h of incubation (Figure 2A; *p* < 0.001).

### 2.3. Heat Shock Protein 70 (HSP70) Concentrations

**Figure 3:**
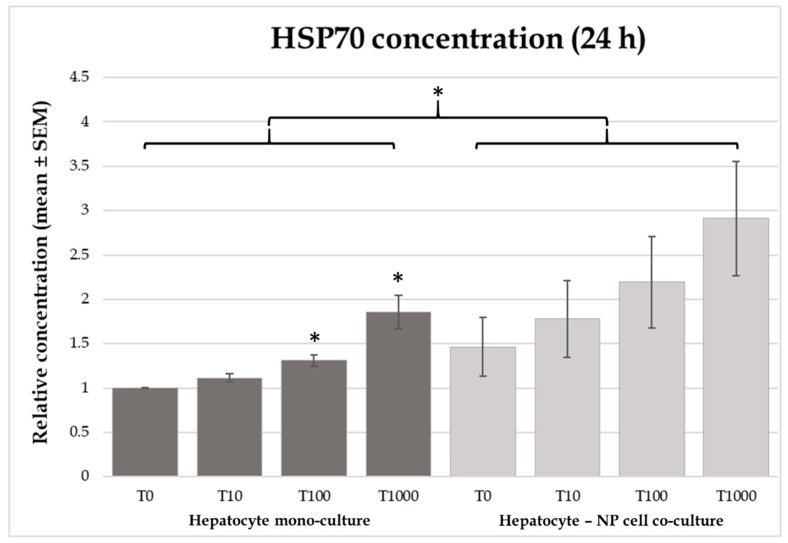
Effects of T-2 toxin treatment on HSP70 production of chicken-derived primary hepatocyte mono- and hepatocyte—nonparenchymal cell co-cultures with the cell ratio of 6:1 (hepatocytes to nonparenchymal cells). Cells after 24 h culturing were treated with different concentrations of T-2 toxin for 8 or 24 h. “T0” refers to control group (without T-2 toxin treatment); “T10” refers to 10 nmol/L T-2 toxin treatment; “T100” refers to 100 nmol/L T-2 toxin treatment; T1000 refers to 1000 nmol/L T-2 toxin treatment. HSP70 concentrations were measured by chicken specific ELISA tests. Relative concentrations were calculated by considering the mean value of T0 hepatocyte mono-cultures as 1. Results are expressed as mean ± SEM. * *p* < 0.05. n = 3/group.

Higher (*p* = 0.025) HSP70 concentrations were found in the culture media of co-cultures compared to those of the hepatocyte mono-culture models (Figure 3). Due to methodological difficulties, no analysable data is available for HSP70 after 8 h incubation. Addition of T-2 toxin at 100 or 1000 nmol/L elevated HSP70 concentrations (*p* = 0.0389 and *p* = 0.0444) in the hepatocyte mono-cultures after 24 h incubation. However, no differences were detected in the co-cultures (Figure 3).

### 2.4. Interleukin-6 and -8 Concentrations

**Figure 4:**
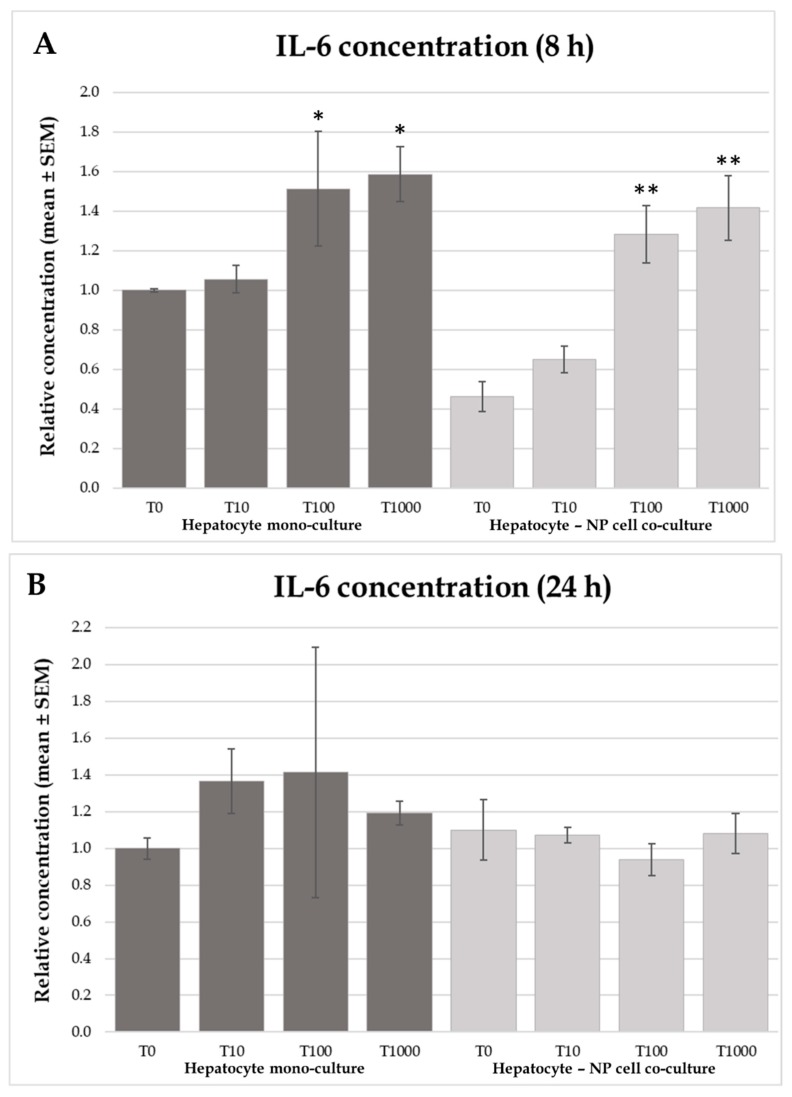
Effects of T-2 toxin treatment on HSP70 production of chicken-derived primary hepatocyte mono- and hepatocyte—nonparenchymal cell co-cultures with the cell ratio of 6:1 (hepatocytes to nonparenchymal cells). Cells after 24 h culturing were treated with different concentrations of T-2 toxin for 8 (**A**) or 24 h (**B**). “T0” refers to control group (without T-2 toxin treatment); “T10” refers to 10 nmol/L T-2 toxin treatment; “T100” refers to 100 nmol/L T-2 toxin treatment; T1000 refers to 1000 nmol/L T-2 toxin treatment. HSP70 concentrations were measured by chicken specific ELISA tests. Relative concentrations were calculated by considering the mean value of T0 hepatocyte mono-cultures as 1. Results are expressed as mean ± SEM. * *p* < 0.05; ** *p* < 0.01. n = 3/group.

The IL-6 concentration differed in both culture types when comparing 100 nmol/L or 1000 nmol/L T-2 toxin supplemented groups to the control after 8 h of incubation treatment in both hepatocyte mono-cultures and hepatocyte—NP cell co-cultures (hepatocyte mono-culture: *p* = 0.0465; *p* = 0.0153 for 100 and 1000 nmol/L T-2 toxin, respectively; co-culture: *p* = 0.00752; *p* = 0.00605 for 100 and 1000 nmol/L T-2 toxin, respectively). In the meanwhile, IL-6 concentrations were unchanged after 24 h of incubation time (Figure 4).

**Figure 5:**
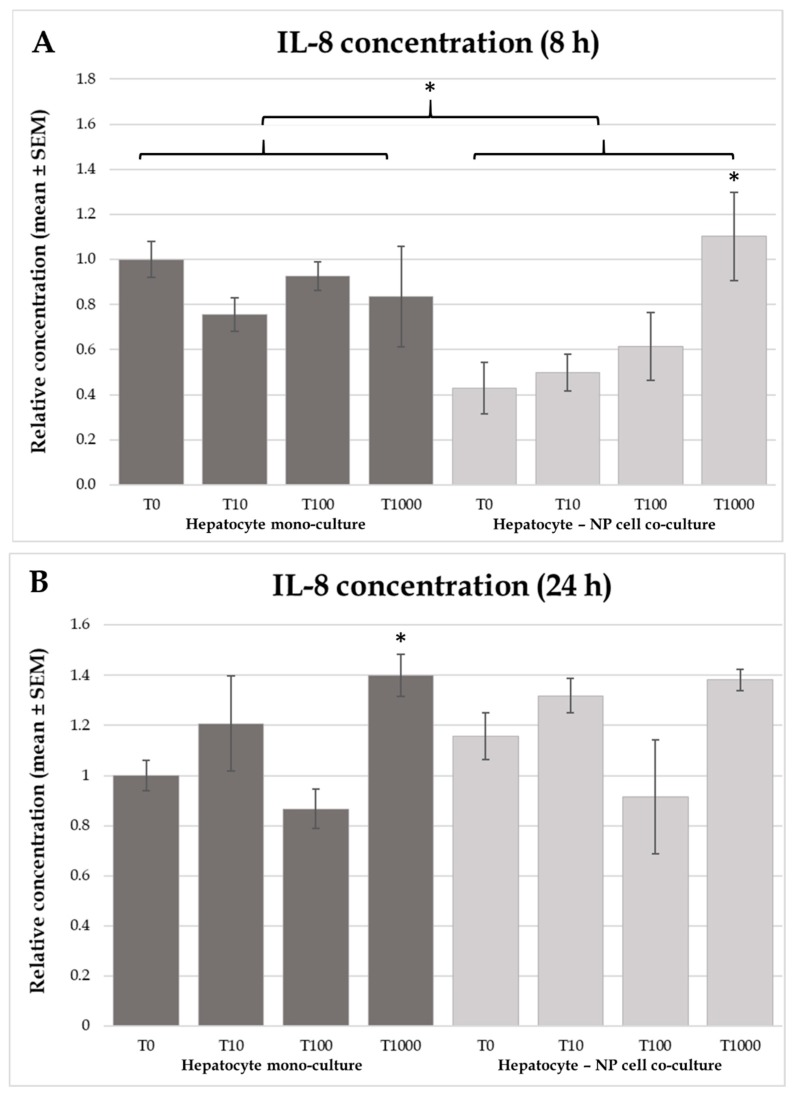
Effects of T-2 toxin treatment on IL-8 production of chicken-derived primary hepatocyte mono- and hepatocyte—nonparenchymal cell co-cultures with the cell ratio of 6:1 (hepatocytes to nonparenchymal cells). Cells after 24 h culturing were treated with different concentrations of T-2 toxin for 8 (**A**) or 24 h (**B**). “T0” refers to control group (without T-2 toxin treatment); “T10” refers to 10 nmol/L T-2 toxin treatment; “T100” refers to 100 nmol/L T-2 toxin treatment; T1000 refers to 1000 nmol/L T-2 toxin treatment. IL-8 concentrations were measured by chicken specific ELISA tests. Relative concentrations were calculated by considering the mean value of T0 hepatocyte mono-cultures as 1. Results are expressed as mean ± SEM. * *p* < 0.05; n = 3/group.

In the co-culture model higher IL-8 concentration was shown, in the culture media of cells treated with 1000 nmol/L T-2 toxin than in controls incubated for 8 h (*p* = 0.0479), no such significant effect was found in the hepatocyte mono-culture model (Figure 5A). In contrast, 24 h incubation with 1000 nmol/L toxin concentration resulted in higher IL-8 levels (*p* = 0.018) in the hepatocyte mono-culture model. However, 24 h toxin incubation did not affect IL-8 levels in the co-cultures (Figure 5B). Concerning the different models, higher IL-8 production (*p* = 0.00677) was shown in the hepatocyte mono-cultures after 8 h incubation but not after 24 h (Figure 5A).

## 3. Discussion

Different chicken-derived primary cell culture models of hepatic origin were successfully established and characterized by our research group previously [24]. In contrast to mammals, no such primary hepatic cell culture model exists. In the present study the cellular effects of T-2 toxin have been investigated in hepatocyte mono-culture and hepatocyte—NP cell co-culture models. Based on our recent results, the gained NP cell fraction consisted of mainly macrophages, predominantly Kupffer cells (resident hepatic macrophages). Our established model can be suitable to assess the specific role of different liver-derived cell types using 6:1 ratio (hepatocyte to NP cells), which refers to a milder hepatic inflammatory state representing a moderate intrahepatic macrophage migration.

Concerning the major effects of T-2 toxin, many studies can be found in the literature; however, the results are often conflicting. This inconsistency may originate from the dissimilar study designs and the various applied T-2 toxin concentrations, or it could be explained by the species-specific differences as well. Therefore, to clarify the questions related to this scientific field the aim in this study was to investigate the effects of T-2 toxin on the cellular metabolic activity and furthermore on the oxidative and inflammatory status of both hepatocyte mono-cultures and hepatocyte—NP cell co-cultures.

The incubation circumstances and the applied T-2 toxin concentrations were determined based on our pilot studies and on literature data. According to this, T-2 toxin was applied in 10, 100, 1000 nmol/L concentrations in the culture medium, similarly to another chicken related hepatic in vitro experiment carried out by Yang et al. [25].

In the present study, T-2 toxin treatment decreased the cellular metabolic activity in all of the applied concentrations in both of the cell culture models. This result is in correlation with the findings that trichothecene mycotoxins inhibit protein synthesis, and are able to cause severe damage on mitochondrial membranes and on endoplasmatic reticulum resulting in morphological and functional impairments [26]. Since T-2 toxin can bind to different proteins, it decreases the activity of various enzymes, such as succinate dehydrogenase which plays a crucial role in essential catabolic pathways of the cells. This results in cellular energy deficiency by decreasing the rate of the citric acid cycle and by consequently that of the respiratory chain [27,28].

Apart from the DNA- and membrane-related damage, another important harmful effect caused by trichothecene mycotoxins is the generation of reactive oxygen species and consequently appearing oxidative stress. Oxidative stress induced by T-2 toxin was earlier investigated by in vitro [11] and in vivo studies [29]. In the literature, reports can be found in correlation with the in vitro oxidative effects of the T-2 toxin in different cell types such as Vero cells [30], porcine ovarian granulosa cells [31], and human monocytes [32]. Studies were also carried out on human hepatoma cells [33] and chicken primary hepatocyte cell cultures [25]; however, regarding the exact hepatic effects caused by T-2 toxin—especially in avian species—and the role of hepatocytes or liver-derived macrophages concerning the mechanism of action is still unknown.

In the actual study no differences were found regarding the effects of T-2 toxin on extracellular ROS (H_2_O_2_) concentration either in the hepatocyte mono-culture or in the hepatocyte—NP cell co-culture models. In addition, differences were observed in the cellular metabolic activity, interleukin production, and HSP70 levels at the same time. It is also worth mentioning, that in several trichothecene toxin related studies carried out in broiler chicken DNA damage and apoptosis were described, whereas oxidative stress was not observed measuring ROS levels and the concentration of different lipid peroxidation markers in human colon carcinoma cells in vitro [16,17,34]. Therefore, it has been suggested, that oxidative damage is probably not the main contributive factor to trichothecene mycotoxin (T-2 and DON) toxicity in chicken. Comparing the two applied cell culture models, lower H_2_O_2_ concentration was found in the co-cultures independently of the T-2 toxin treatment. Bozem et al. (2018) showed, that real time extracellular H_2_O_2_ concentration was affected by several extra- and intracellular mechanisms, such as the production and degradation intensity of the molecule [35]. In the study of Spolarics et al. (1996), increased H_2_O_2_ breakdown was reported in endotoxin challenged Kupffer cells as a protective mechanism against phagocytosis related oxidative stress [36].

Heat shock proteins show correlation with the cytoprotective mechanisms against different stressors and toxic effects. This protective mechanism is presumably in correspondence with the prevention of protein aggregation and other protein modifications [37]. Another possible mechanism of action can be the enhancement of cellular protein and antioxidant stability, preventing the oxidative injuries caused by different noxious agents [38]. In our study a significant increase was found in the HSP70 concentrations of culture media after 24 h of 100 nmol/L and 1000 nmol/L T-2 toxin incubation in hepatocyte mono-cultures. This finding is in correspondence with other in vitro studies carried out on Vero cells [20]. In spite of these findings, Bouaziz et al. (2013) observed increased HSP70 protein level in case of Vero cells treated with T-2 toxin, only in combination with zearalenol, but not alone [33]. Similarly, the trichothecene mycotoxin DON did not influence HSP70 in vitro, despite its general harmful effects [16]. The effects of mycotoxins on HSP70 production can be refined by species-specific differences as well. Up to date, there is no data in the literature regarding the effects of T-2 toxin on HSP70 production in chicken.

Concentration of pro-inflammatory cytokine IL-6 was elevated as a result of both 100 nmol/L and 1000 nmol/L T-2 toxin treatment in the hepatocyte mono-culture, as well as the hepatocyte—NP cell co-culture models after 8 h incubation time as a consequence of the rapid toxin attack. However, in the cell culture models treated with T-2 toxin for 24 h—corresponding to a longer toxin exposure accompanied with an adaptation response—no significant differences were found. These results are similar to those described by Wang et al. (2012) and Wu et al. (2014) [39,40]. These latter studies have shown a significant upregulation in the mRNA expression of different inflammatory factors such as IL-1β, IL-6, and TNF-α in RAW264.7 murine macrophages in dose-dependent manner. Similarly, a significant increase in IL-6 mRNA expression and serum IL-6 cytokine levels were reported in rats in vivo, treated by T-2 toxin [41]. Correspondingly to the above mentioned results, Fu et al. (2001) observed a similarly higher IL-6 level after T-2 treatment measured from the cell culture media of in vitro cultured human foetal chondrocytes [42].

In the IL-8 concentration significant differences were found after 8 h of incubation time in the co-culture models, while in the case of hepatocyte mono-cultures significantly higher IL-8 concentration was only found after 24 h of incubation time. These differences were shown in both cases in the 1000 nmol/L T-2 toxin treated groups compared to controls. Investigations carried out on Caco-2 cell lines, demonstrated a concentration dependent significant increase of IL-8 [43]. It was already described that trichothecenes could activate the mitogen-activated protein kinase (MAPK) pathway, resulting in the upregulation of pro-inflammatory cytokines, contributing to functional disorders and apoptosis of the cells [13].

When comparing the applied cell culture models, significant differences were found in extracellular H_2_O_2_, HSP70, and IL-8 levels, but the cellular response of various culture types to T-2 toxin did not significantly differ from each other. The concentration of these molecules in cell culture supernatant is determined by their cellular synthesis, release, utilization, and breakdown, highly depending on the comprising cell types and the influencing factors (concentration and duration of T-2 toxin exposure). Based on our results, the observed differences between mono- and co-cultures cannot be well explained, but the critical role of the comprising cell types should be emphasized.

General harmful effects of T-2 toxin were already widely investigated, although there are numerous unanswered questions regarding certain cellular actions in chicken. Based on our results, the established primary hepatocyte mono-cultures and hepatocyte—NP cell co-cultures derived from chicken were found to be proper models to study the specific molecular effects of T-2 toxin. The toxin could strongly diminish the function of chicken liver cells, reflected by decreased metabolic rate, and trigger an inflammatory response by increasing pro-inflammatory cytokine and HSP70 production. However, no changes were found in the extracellular H_2_O_2_ levels, which can suggest that ROS production may not play a key mediatory role in the cytotoxic effects of T-2 toxin on chicken liver. In conclusion, the present study provided novel data concerning the hepatic action of T-2 toxin, highlighting the molecular mechanisms and emphasizing the potential hazards of T-2 toxin in poultry farming.

## 4. Materials and Methods

All reagents used in our study were purchased from Sigma-Aldrich (Darmstadt, Germany) except when otherwise specified. Animal procedures described hereinafter were performed in strict accordance with the international and national law along with institutional guidelines and were confirmed by the Local Animal Welfare Committee of the University of Veterinary Medicine, Budapest and by the Government Office of Pest County, Food Chain Safety, Plant Protection and Soil Conservation Directorate, Budapest, Hungary (permission number: PEI/001/1430-4/2015, approval date: 27 April 2015).

### 4.1. Isolation and Culturing of Hepatocytes and Nonparenchymal Cells

Isolation of hepatocytes and nonparenchymal cells (NP cells) was performed from three weeks old Ross 308 male broiler chickens obtained from Gallus Poultry Farming and Hatching Ltd, Devecser, Hungary. Animals were reared and fed according to the recommendations of the breeder [44]. After decapitation of the animal in CO_2_ narcosis and following the cannulation of the gastropancreaticoduodenal vein, liver was flushed and exsanguinated through the hepatic portal system with different buffer solutions, using a multistep in situ perfusion. First, liver was perfused by 150 mL Hanks’ balanced salt solution (HBSS), containing 0.5 mmol/L ethylene glycol tetraacetic acid (EGTA). Thereafter, liver was flushed by 150 mL EGTA-free HBSS, followed by 100 mL MgCl_2_ and CaCl_2_ (both 7 mmol/L) containing HBSS supplemented with 1 mg/mL type IV collagenase (Nordmark, Uetersen, Germany). HBSS was supplemented with 0.035% NaHCO_3_ during every phase. Collagenase-mediated digestion of liver tissue was applied in order to disintegrate the organ and efficiently release the cells. Velocity of buffer circulation was set up to 30 mL/min during the whole process. During the perfusion of the liver, all perfusion buffers were warmed up to 40 °C and oxygenated by Carbogen (95% O_2_, 5% CO_2_).

After excision and gentle shaking by forceps, liver was placed for 45 min into 25 mg/mL bovine serum albumin (BSA) containing ice-cold HBSS. Based on the previous experiences of our research group, this incubation step is effective in avoiding undesired cluster formation.

Hepatocyte and NP cell enriched fractions were isolated using multistep differential centrifugation. First, cell suspensions were centrifuged three times at low speed (100× *g*) for 3 min in Williams’ medium E supplemented with 0.22% NaHCO_3_, 50 mg/mL gentamycin, 2 mM glutamine, 4 µg/L dexamethasone, 20 IU/L insulin, and 5% foetal bovine serum (FBS). Between these three steps, NP cells (predominantly Kupffer cells) containing supernatants were collected separately and hepatocyte enriched pellets were always freshly resuspended in cell culture medium. After the last resuspendation, purified hepatocyte fraction was received.

The beforehand collected supernatants were also centrifuged at 350× *g* for 10 min to remove red blood cells and remaining hepatocytes. The supernatant was again centrifuged at 800× *g* for 10 min, and the sediment was resuspended in a culture medium. Cell suspension, gained after these purification steps contained the NP cells enriched fraction. The viability of both hepatocytes and NP cells was tested by trypan blue exclusion test and exceeded 90% in both cases. Cell yield was inspected by cell counting in Bürker’s chamber to adjust the appropriate cell concentrations (hepatocyte mono-cultures: 10^6^ cells/mL; co-cultures: 8.5 × 10^5^ cells/mL hepatocytes, 1.5 × 10^5^ cells/mL NP cells).

Hepatocytes were seeded onto 96- and 6-well Greiner Advanced TC cell culture dishes (Greiner Bio-One Hungary Kft., Mosonmagyaróvár, Hungary) coated with collagen type I (10 μg/cm^2^). Regarding the co-cultures, the same type of collagen-coated culture dishes was used as for mono-cultures, and in the first step, NP cells were seeded for 20 min incubation time. After the successful attachment to the culture dish surface, the medium was removed and hepatocytes were seeded. The cell ratio in co-cultures was set to 6:1 (hepatocytes to NP cells). Seeding volume was 1.5 mL/well on 6-well plates, 100 µL/well on 96-well plates.

Hepatocyte mono-cultures and hepatocyte—NP cell co-cultures were incubated at 38.5 °C with 5% CO_2_ in Williams’ medium E, supplemented as described previously. Culture medium was changed 4 h after plating. Culture media contained 5% FBS only during the first 24 h of culturing. Confluent monolayer mono- and co-cultures were gained after 24 h of incubation.

### 4.2. Treatments and Measurements

Cell cultures were challenged to T-2 toxin in different concentrations. Williams’ medium E was supplemented with 0 (control), 10, 100, or 1000 nmol/L T-2 toxin. Treatment with toxin containing media lasted either for 8 or for 24 h, respectively, in all of the applied cell culture models. Later, samples were taken from culture media of the 6-well plates after both incubation times and cells were lysed using Mammalian Protein Extraction Reagent (M-PER^TM^, Thermo Fisher Scientific, Waltham, MA, USA) supplemented with 1% Halt Protease Inhibitor Cocktail (Thermo Fisher Scientific, Waltham, MA, USA) and 1% ethylene diamine tetraacetic acid (EDTA). Samples were stored until further analysis at −80 °C.

Following the T-2 toxin challenge, metabolic activity of cells seeded onto 96-well plates was monitored by CCK-8 assay. The test was prepared according to the manufacturer’s protocol. By the assay, the amount of NADH+H^+^ produced in the catabolic reactions of the cells was monitored. First, 10 µL CCK-8 reagent and 100 µL Williams’ medium E were given to the cultured cells, and the absorbance was measured at 450 nm with a Multiskan GO 3.2 reader (Thermo Fisher Scientific, Waltham, MA, USA) after 2 h of incubation at 38.5 °C.

Extracellular H_2_O_2_ concentration was determined in the culture medium using Amplex Red method (Thermo Fisher Scientific, Waltham, MA, USA). In the assay, the applied substrate reacts with H_2_O_2_, producing fluorescent resorufin. After 30 min incubation at room temperature of 50 µL freshly prepared, Amplex Red (100 µM) and HRP (0.2 U/mL) containing working solution and 50 µL culture medium, fluorescence was detected with a Victor X2 2030 fluorometer (λ_ex_ = 560 nm; λe_m_ = 590 nm, Perkin Elmer, Waltham, MA, USA).

Production of HSP70, IL-6, and IL-8 was measured by chicken-specific ELISA tests (MyBioSource, San Diego, CA, USA) in the collected medium. In order to standardize values gained from the previously mentioned methods, total protein concentration of cell lysates was determined by Pierce BCA Protein Assay (Thermo Fisher Scientific, Waltham, MA, USA).

### 4.3. Statistics and Calculations

All the data analysis was performed using R 3.5.3. software (GNU General Public License, Free Software Foundation, Boston, MA, USA). On 96-well plates, six wells were included in one treatment group, while cells were examined in triplicates on 6-well plates. Differences between various groups were assessed using one-way analysis of variance (ANOVA) and post-hoc tests for pairwise comparisons. All data were standardized to protein concentrations measured from the cell lysates. Results were assessed as the mean ± standard error of the mean (SEM). Differences were assumed significant at *p* < 0.05.
